# A cross-sectional analysis of publication of pediatric global health abstracts from seven major international conferences

**DOI:** 10.1371/journal.pgph.0002523

**Published:** 2023-10-25

**Authors:** Catherine Shari, Tory Prynn, Sarah Mohammedahmed Abbas, Tommy Davis, Jeesoo Lee, Gandolina Melhem, Hussein K. Manji, Brittany L. Murray, Richard Omore, Shayli Patel, Stephanie J. Sirna, Adrianna L. Westbrook, Chidiebere V. Ugwu, Sabira A. Versi, Karim P. Manji, Chris A. Rees

**Affiliations:** 1 Emergency Medicine Department, Muhimbili National Hospital-Mloganzila, Dar es Salaam, United Republic of Tanzania; 2 Department of Pediatrics, Emory University School of Medicine, Atlanta, Georgia, United States of America; 3 Independent Researcher, Khartoum, Sudan; 4 Emory University, Atlanta, Georgia, United States of America; 5 Emory University School of Medicine, Atlanta, Georgia, United States of America; 6 University of Pennsylvania School of Medicine, Philadelphia, Pennsylvania, United States of America; 7 Department of Emergency Medicine, Muhimbili University of Health and Allied Sciences, Dar es Salaam, United Republic of Tanzania; 8 Accident and Emergency Department, Aga Khan Hospital Dar es Salaam, Dar es Salaam, United Republic of Tanzania; 9 Department of Emergency Medicine, Emory University School of Medicine, Atlanta, Georgia, United States of America; 10 Emergency Medicine, Children’s Healthcare of Atlanta, Atlanta, Georgia, United States of America; 11 Kenya Medical Research Institute, Center for Global Health Research, (KEMRI-CGHR), Kisumu, Kenya; 12 Department of Medicine, Weill Cornell Medicine, New York, New York, United States of America; 13 Pediatric Biostatistics Core, Department of Pediatrics, Emory University School of Medicine, Atlanta, Georgia, United States of America; 14 Intensive Care Unit, The Aga Khan Hospital, Dar-es-salaam, United Republic of Tanzania; 15 Department of Paediatrics and Child Health, Muhimbili University of Health and Allied Sciences, Dar es Salaam, United Republic of Tanzania; Baylor College of Medicine, UNITED STATES

## Abstract

Research presented at conferences may increase context-specific evidence in low- and middle-income countries (LMICs), where global childhood disease burden is greatest and where massive relative deficits in research persist. Publication of studies presented at conferences is necessary for complete results dissemination. Our objective was to determine the frequency of publication of pediatric global health conference abstracts and to identify factors associated with publication. We conducted a cross-sectional study of abstracts that reported pediatric research conducted in at least one LMIC presented at seven major scientific conferences in 2017, 2018, and 2019. We used PubMed, EMBASE and Google Scholar to search for publications of the results presented as abstracts. We created a Kaplan-Meier curve to determine the cumulative incidence of publications and used predetermined abstract-level factors to create a multivariable Cox proportional hazard model to identify factors associated with time to publication. There were 8,105 abstracts reviewed and 1,433 (17.7%) reported pediatric research conducted in one or more LMICs. The probability of publication of pediatric global health abstracts was 33.6% (95% confidence interval [CI] 31.2–36.1%) at 24 months and 46.6% (95% CI 44.0–49.3%) at 48 months. Abstracts that reported research conducted in East Asia and Pacific (adjusted hazard ratio [aHR] 3.06, 95% CI 1.74–5.24), South Asia (aHR 2.25, 95% CI 1.30–3.91%), and upper-middle-income countries (1.50, 95% CI 1.12–2.02) were published sooner than those that reported research in LMICs in Europe and Central Asia and lower-middle-income countries, respectively. Fewer than half of pediatric global health abstracts were published in peer-reviewed journals up to four years after presentation at international conferences. Efforts are urgently needed to promote the widespread and long-lasting dissemination of pediatric research conducted in LMICs presented as abstracts to provide a more robust evidence base for both clinical care and policy related to child health.

## Introduction

Most global childhood disease burden, including disability, morbidity, and mortality, lies in low- and middle-income countries (LMICs) [1]. However, there are massive relative deficits in the amount of research conducted in LMICs especially addressing childhood disease burden [2–5]. Although there has been a recent increase in publications reporting work conducted in LMICs, the amount of published work originating in high-income countries (HICs) has greatly surpassed any relative gains in published work from LMICs [6].

Articles published in peer-reviewed journals are used by clinicians and policy makers as a source of evidence to develop treatment guidelines, standards of practice, and to shape health policies. As most publications report work conducted in HICs [6], clinicians and policy makers in LMICs often rely on research conducted in settings unlike their own. However, results from such publications may not be directly applicable or feasible in resource-limited settings. Peer-reviewed publications are especially necessary for broad dissemination of findings to foster further understanding of how to reduce suffering in high-disease burden regions. Furthermore, peer-reviewed publications help academicians advance their careers and become competitive for funding and additional research opportunities [7]. With the relative paucity of published research conducted in LMICs, it is of utmost importance that research conducted in these settings result in peer-reviewed publications.

Research presented at global health conferences, which undergoes peer-review and requires acceptance prior to presentation, is a potential body of research with results that may increase context-specific evidence generated in LMICs. However, the frequency of abstracts presented at global health conferences resulting in peer-reviewed publication is unknown. In studies of registered clinical trials, results from 25–35% of trials typically remain unpublished several years after trial completion [8]. In general, approximately half of abstracts presented at major conferences result in peer-reviewed publications years after conferences take place [9, 10]. However, prior studies have not specifically assessed publication rates of pediatric global health abstracts.

Here, our objective was to determine the frequency of publication of presented pediatric global health abstracts and to identify factors associated with time to publication. Such an understanding has the potential to inform efforts to reduce the frequency of nonpublication of pediatric global health abstracts, which can promote the widespread and long-lasting dissemination of pediatric research conducted in LMICs, which, in turn, has the potential to influence clinical care and policy.

## Materials and methods

### Study design

We conducted a cross-sectional study of abstracts that reported pediatric research conducted in at least one LMIC presented at seven major scientific conferences held in 2017, 2018, and 2019. We selected this time period to provide at least 2.5 years and up to 5.5 years between the conference date and our search for peer-reviewed publications reporting the work previously reported at a scientific conference. Countries were classified as low-income, lower-middle income, upper-middle income, and high-income according to the World Bank classification schema [11]. The Emory University Institutional Review Board exempted this study from review because all data were publicly available in conference abstract booklets and peer-reviewed publications and no primary patient data were collected.

### Conference selection

We included pediatric global health abstracts from American Society of Tropical Medicine and Hygiene, Pediatric Academic Societies, American Academy of Pediatrics National Conference & Exhibition, Excellence in Pediatrics, Annual Academic International Medicine Congress, Canadian Pediatric Society, and the European Academy of Paediatrics conferences. These conferences were selected because they are major international global health or pediatrics conferences, included pediatric presentations, included presentations on a variety of topics presented by investigators from around the world, and had abstracts or presentation titles and author lists that were accessible from the included years. We attempted to include regional pediatric conferences from Latin America, sub-Saharan Africa, and South Asia, but we could not identify conferences with available abstract booklets from the included years so limited our conferences to the seven listed above.

### Data extraction and variables

We reviewed the title and content of all abstracts presented at the included conferences. We included abstracts that reported research conducted in at least one LMIC and exclusively included participants aged 0–18 years, or those in which the median or mean age of participants was ≤18 years per prior studies [12, 13]. Abstracts that did not meet these criteria were excluded. We developed a REDCap form to extract the candidate variables of interest from each included abstract [14].

We extracted the following variables from included abstracts: conference year, abstract title, number of authors, number of authors affiliated with the country where the study was conducted, first and senior (i.e., last) author country affiliations, study country name(s), number of study countries, study design, if abstract reported the results of a program, disease(s) studied, sample size, and, where available, type of presentation (i.e., abstract only, poster, platform, or plenary presentation). Diseases studied were categorized using the Institute of Health Metrics and Evaluation (IHME) Level 2 conditions [15].

### Publication identification

We used the search engines PubMed, EMBASE and Google Scholar to search for publications reporting the aims of the included abstracts. We used the methodology established by Nigrovic SE, et al. [10] to identify publications that reported the results of abstracts at the included scientific conference. Specifically, we considered a peer-reviewed publication to be associated with the conference abstract if 1) the abstract title was the same and the author bylines included the same first and last author or 2) the first and last authors of the abstract were co-authors on a peer-reviewed manuscript that reported the same objectives, study design, and disease studied as the abstract presented. For peer-reviewed publications, we collected the journal name and date of publication on matching peer-reviewed publications.

For abstracts in which an associated peer-reviewed publication was not identified, a second reviewer independently searched for a publication. If no publication was identified by either reviewers, an email was sent to either the first or last author of the abstract to inquire if there was a publication reporting the aims of the presented abstract. All questions regarding the potential match between an abstract and a publication were discussed with the senior author of this manuscript. A final review was conducted on November 15, 2022.

### Statistical analyses

We conducted descriptive statistics for characteristics of pediatric global health abstracts. We compared the proportion of abstracts that were published up to 5.5 years after the conference date by diseases studied using the Chi-square or Fisher’s test. We created a Kaplan-Meier curve to determine the cumulative incidence of publications associated with pediatric global health abstracts presented at the included conferences. Publications were censored if not published by November 15, 2022. We created a multivariable Cox proportional hazard model to test the association of candidate variables and their relation to the outcome of publication. Candidate variables were selected based on availability in the included abstracts, the results of prior studies assessing abstract publication rates [9], and factors that were theoretically thought to be associated with the outcome of later publication in peer-reviewed journals. Additionally, given the results of previous studies [9], we conducted a *post hoc* analysis to assess if abstracts that reported research conducted in primarily English-speaking countries were more likely to be published than those reporting work conducted in countries where another language is the primary language. All statistical analyses were conducted using SAS 9.4 (Cary, NC) and figures were created in R v4.1.3 (R Core Team 2022; Vienna, Austria).

## Results

**Fig 1:**
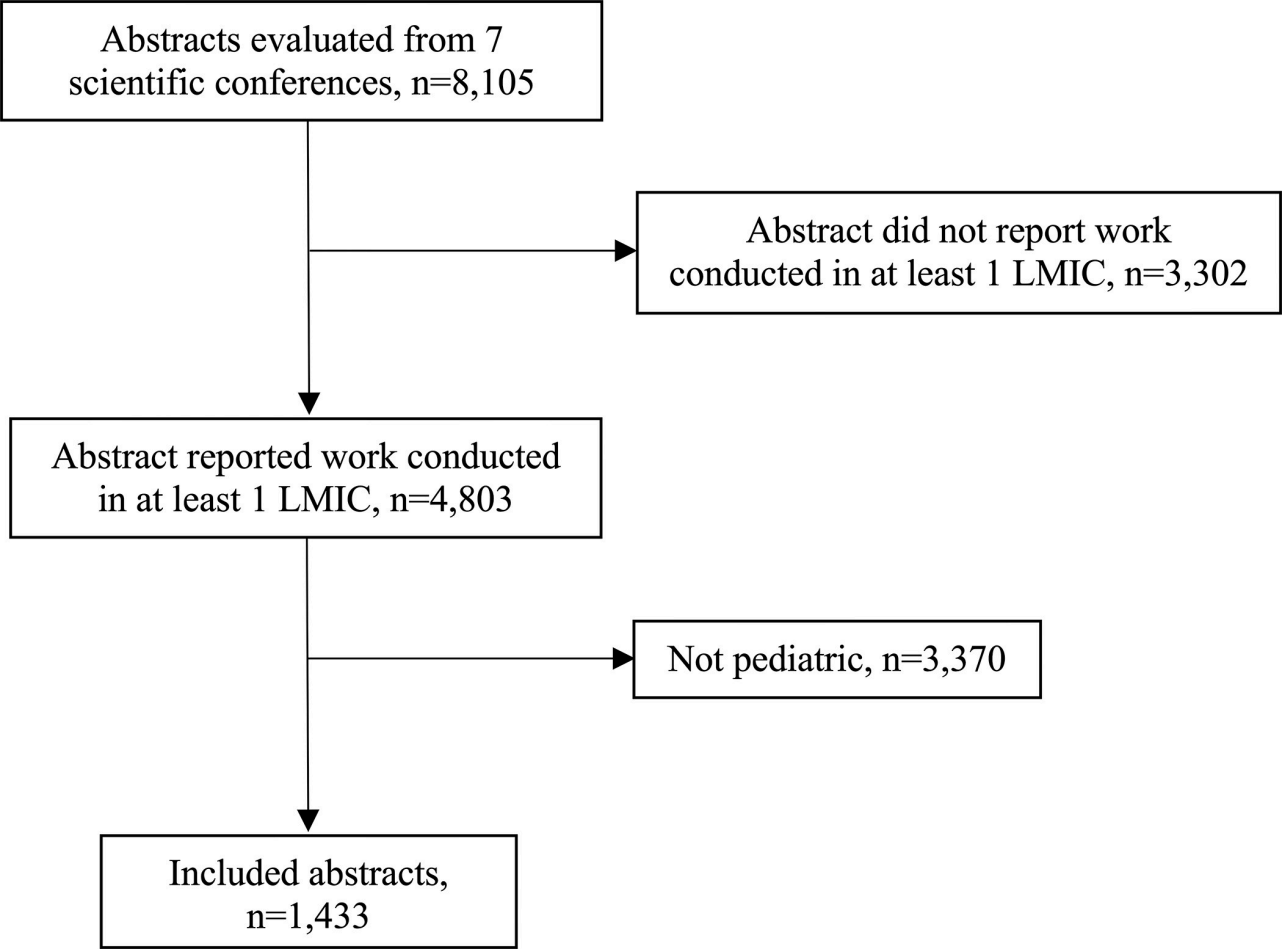
PRISMA diagram for selection of pediatric abstracts that reported research conducted in low- and middle-income countries.

There were 8,105 abstracts in the booklets of the included conferences and 1,433 (17.7%) reported pediatric research conducted in at least one LMIC (Fig 1). The majority of included abstracts were presented at conferences held in the United States (81.0%, n = 1,161) (Table 1). Over half of the abstracts reported research conducted in sub-Saharan Africa (50.9%, n = 729) and 42.8% (n = 613) reported research conducted in lower-middle income countries. There was a median of 7 authors per abstract (interquartile range [IQR] 4–10). Cross-sectional studies were the most common (42.8%, n = 614) study design employed and 30.2% (n = 433) of the pediatric global health abstracts reported results of studies with sample sizes of <100 participants.

**Table 1:** Characteristics of included pediatric abstracts reporting work conducted in low- and middle-income countries (N = 1,433).

Abstract Characteristic	n (%)
**Conference**	
Held in the United States^1^	1,161 (81.0)
Held outside the United States^2^	272 (19.0)
**Year of Presentation**	
2017	581 (40.5)
2018	480 (33.5)
2019	372 (26.0)
**Study countries, n**	
1	1,334 (93.1)
>1	99 (6.9)
**Study country region**	
East Asia and Pacific	92 (6.4)
Europe And Central Asia	149 (10.4)
Latin America and The Caribbean	188 (13.1)
Middle East and North Africa	33 (2.3)
Multi-region	51 (3.6)
South Asia	191 (13.3)
Sub-Saharan Africa	729 (50.9)
**Study Country income bracket**	
Upper-middle income	306 (21.4)
Lower-middle income	613 (42.8)
Low income	427 (29.8)
Multi-income	87 (6.1)
**Authors per abstract (median, IQR)**	7 (4, 10)
**Authors per abstract**	
1–5	539 (37.6)
>5	894 (62.4)
**First author affiliated with study country**	
Yes	322 (22.5)
No	1,111 (77.5)
**Senior author affiliated with study country**	
Yes	214 (14.9)
No	1,219 (85.1)
**Study Design**	
Case control	80 (5.6)
Case series	64 (4.5)
Cohort study	368 (25.7)
Cross-sectional	614 (42.8)
Qualitative study	57 (4.0)
Quality improvement	31 (2.2)
Randomized controlled trial	117 (8.2)
Unclear	102 (7.1)
**Description of program**	
Yes	568 (39.6)
No	845 (59.0)
Unclear	20 (1.4)
**Sample size**	
<100	433 (30.2)
100–300	279 (19.5)
>300	708 (49.4)
Unclear	13 (0.9)
**Type of presentation**	
Abstract only	51 (3.6)
Platform/Plenary presentation	45 (3.1)
Poster	385 (26.9)
Unclear	952 (66.4)

^1^Includes Pediatric Academic Societies, American Academy of Pediatrics National Conference & Exhibition, American Society of Tropical Medicine and Hygiene, and Annual Academic International Medicine Congress conferences.

^2^Includes Excellence in Pediatrics, Canadian Pediatric Society, and European Academy of Paediatrics conferences.

After a median follow up of 48 (IQR 42–60) months from the respective conference date, 46.5% (n = 666) of abstracts had full peer-reviewed publications. The majority of these publications (95.3%, n = 635) were identified through our search of PubMed, EMBASE and Google Scholar, and 4.7% (n = 31) were identified through responses of authors to our email queries. We contacted 767 investigators of abstracts for which no corresponding publication was found and 23.1% (n = 177) responded to our email query about publications associated with their abstracts, and 31 additional publications were identified through this correspondence.

Peer-reviewed publications were more common among abstracts that reported research on neglected tropical diseases and malaria and other infectious diseases (Table 2). Conversely, peer-reviewed publications were less common for abstracts that reported research on musculoskeletal disorders, neurological disorders, and oral disorders, though the number of these abstracts was limited.

**Table 2:** Comparison of publication of pediatric global health abstracts by diseases studied.

	Abstracts with Peer-reviewed publications (N = 666), n (column %)	Row %	Abstracts without Peer-reviewed publications (N = 767), n (column %)	Row %	*P* value
Cardiovascular diseases	6 (0.9)	0.6	4 (0.5)	0.4	0.53
Chronic respiratory diseases	4 (0.6)	0.4	6 (0.8)	0.6	0.76
Congenital birth defects	46 (6.9)	38.7	73 (9.5)	61.3	0.07
Diabetes and kidney disease	2 (0.3)	18.2	9 (1.2)	81.2	0.06
Diarrheal diseases	56 (8.4)	46.7	64 (8.3)	53.3	0.97
Digestive diseases	16 (2.4)	45.7	19 (2.5)	54.3	0.93
Endocrine, metabolic, blood, and immune disorders	21 (3.2)	44.7	26 (3.4)	55.3	0.80
Hemoglobinopathies and hemolytic anemias	10 (1.5)	34.4	19 (2.5)	65.6	0.19
HIV/AIDS	13 (2.0)	59.1	9 (1.2)	40.9	0.23
Maternal disorders	2 (0.3)	0.4	3 (0.4)	0.6	1.00
Mental disorders	0 (0.0)	0.0	5 (0.7)	100.0	0.07
Musculoskeletal disorders	1 (0.2)	11.1	8 (1.0)	88.9	**0.04**
Neglected tropical diseases and malaria	225 (33.8)	51.4	213 (27.8)	48.6	**0.01**
Neoplasms	1 (0.2)	12.5	7 (0.9)	87.5	0.08
Neurological disorders	3 (0.5)	15.8	16 (2.1)	84.2	**0.01**
Nutritional Deficiencies	34 (5.1)	37.4	57 (7.4)	62.6	0.07
Oral disorders	0 (0.00)	0.0	7 (0.9)	100.0	**0.02**
Other	111 (16.7)	48.7	117 (15.3)	51.3	0.47
Other infectious diseases	67 (10.1)	55.4	54 (7.0)	44.6	**0.04**
Respiratory infections and tuberculosis	45 (6.8)	52.9	40 (5.2)	47.1	0.22
Self-harm and interpersonal violence	1 (0.2)	12.5	7 (0.9)	87.5	0.08
Skin and subcutaneous diseases	0 (0.0)	0.0	2 (0.3)	100.0	0.50

Pediatric global health abstracts that reported research conducted in multiple income brackets most commonly had associated publications (n = 54/87, 60.9%) and those conducted in upper-middle income countries alone were least commonly published (n = 123/306, 40.2%) (Table 3). Pediatric global health abstracts that reported results from randomized controlled trials were commonly published (n = 82/117, 70.1%) and those reporting case series were least commonly associated with peer-reviewed publications (n = 12/64, 18.8%). Abstracts presented as platform/plenary presentations were most commonly associated with publications (n = 26/45, 57.8%) and those listed only as abstracts were least commonly published (n = 15/51, 29.4%). There was no significant difference in the proportion of abstracts that reported research conducted in primarily English-speaking countries that were published (48.2%, n = 307/637) compared to those that reported research conducted in countries that were not primarily English speaking (44.1%, n = 320/725) or those multi-country studies that were conducted in both English-speaking and non-English speaking countries (54.9%, n = 39/71) (*P* = 0.11).

**Table 3:** Publication and factors associated with time to publication of pediatric abstracts reporting work conducted in low- and middle-income countries.

Abstract Characteristic	Abstracts with Peer-reviewed publications (N = 666), n (%)	Abstracts without Peer-reviewed publications (N = 767), n (%)	Time to Manuscript Publication, Adjusted Hazard Ratio (95% CI)	*P* value
**Conference**				
Held in the United States,^1^ n = 1,161	590 (50.8)	571 (49.2)	*Referent*	
Held outside the United States,^2^ n = 272	76 (27.9)	196 (72.1)	0.68 (0.45, 1.03)	0.07
**Year of Presentation**				
2017, n = 581	263 (45.3)	318 (54.7)	*Referent*	
2018, n = 480	243 (50.6)	237 (49.4)	1.05 (0.88, 1.26)	0.58
2019, n = 372	160 (43.0)	212 (57.0)	1.03 (0.83, 1.27)	0.82
**Study countries, n**				
1, n = 1334	608 (45.6)	726 (54.4)	1.35 (0.65, 2.84)	0.42
>1, n = 99	58 (58.6)	41 (41.4)	*Referent*	
**Study country region**				
East Asia and Pacific, n = 92	54 (58.7)	38 (41.3)	3.06 (1.79, 5.24)	**<0.001**
Europe and Central Asia, n = 149	31 (20.8)	118 (79.2)	*Referent*	
Latin America and The Caribbean, n = 188	82 (43.6)	106 (56.4)	1.27 (0.75, 2.16)	0.37
Middle East and North Africa, n = 33	11 (33.3)	22 (66.7)	1.79 (0.89, 3.61)	0.10
Multi-region, n = 51	31 (60.8)	20 (39.2)	1.61 (0.76, 3.43)	0.21
South Asia, n = 191	99 (51.8)	92 (48.2)	2.25 (1.3, 3.91)	**0.004**
Sub-Saharan Africa, n = 729	358 (49.1)	371 (50.9)	1.73 (1.00, 2.99)	0.05
**Study Country income bracket**				
Upper-middle income, n = 306	123 (40.2)	183 (59.8)	1.50 (1.12, 2.02)	**0.01**
Lower-middle income, n = 613	274 (44.7)	339 (55.3)	*Referent*	
Low income, n = 427	216 (50.6)	211 (49.4)	1.15 (0.93, 1.43)	0.20
Multi-income, n = 87	53 (60.9)	34 (39.1)	2.29 (0.99, 5.26)	0.05
**Authors per abstract**				
1–5, n = 539	199 (36.9)	340 (63.1)	0.80 (0.67, 0.96)	**0.02**
>5, n = 894	467 (52.2)	427 (47.8)	*Referent*	
**First author affiliated with study country**				
Yes, n = 322	155 (48.1)	167 (51.9)	*Referent*	
No, n = 1,111	511 (46.0)	600 (54.0)	1.17 (0.96, 1.43)	0.12
**Senior author affiliated with study country**				
Yes, n = 214	107 (50.0)	107 (50.0)	*Referent*	
No, n = 1,219	559 (45.9)	660 (54.1)	0.99 (0.79, 1.25)	0.95
**Study Design**				
Cross-sectional, n = 614	267 (43.5)	347 (56.5)	*Referent*	
Case control, n = 80	38 (47.5)	42 (52.5)	1.06 (0.75, 1.50)	0.74
Case series, n = 64	12 (18.8)	52 (81.3)	0.47 (0.26, 0.87)	**0.02**
Cohort study, n = 368	193 (52.4)	175 (47.6)	1.29 (1.07, 1.56)	**0.01**
Qualitative study, n = 57	30 (52.6)	27 (47.4)	1.20 (0.82, 1.77)	0.35
Quality improvement, n = 31	10 (32.3)	21 (67.7)	0.92 (0.47, 1.79)	0.80
Randomized controlled trial, n = 117	82 (70.1)	35 (29.9)	1.70 (1.31, 2.20)	**<0.001**
Unclear, n = 102	34 (33.3)	68 (66.7)	0.74 (0.51, 1.07)	0.11
**Description of program**				
Yes, n = 568	237 (41.7)	331 (58.27)	*Referent*	
No, n = 845	422 (49.9)	423 (50.06)	1.14 (0.95, 1.36)	0.16
Unclear, n = 20	7 (35.0)	13 (65.0)	0.67 (0.31, 1.44)	0.31
**Sample size**				
<100, n = 433	186 (43.0)	247 (57.0)	*Referent*	
100–300, n = 279	135 (48.4)	144 (51.6)	1.01 (0.8, 1.26)	0.97
>300, n = 708	342 (48.3)	366 (51.7)	0.88 (0.73, 1.07)	0.19
Unclear, n = 13	3 (23.1)	10 (76.9)	0.35 (0.11, 1.12)	0.08
**Type of presentation**				
Abstract only, n = 51	15 (29.4)	36 (70.6)	0.57 (0.33, 0.98)	**0.04**
Platform/Plenary presentation, n = 45	26 (57.8)	19 (42.2)	1.16 (0.77, 1.74)	0.48
Poster, n = 385	140 (36.4)	245 (63.6)	0.97 (0.76, 1.23)	0.78
Unclear, n = 952	485 (51.0)	467 (49.1)	*Referent*	

^1^Includes Pediatric Academic Societies, American Academy of Pediatrics National Conference & Exhibition, American Society of Tropical Medicine and Hygiene, and Annual Academic International Medicine Congress conferences.

^2^Includes Excellence in Pediatrics, Canadian Pediatric Society, and European Academy of Paediatrics conferences.

### Time to publication of pediatric global health abstracts

**Fig 2:**
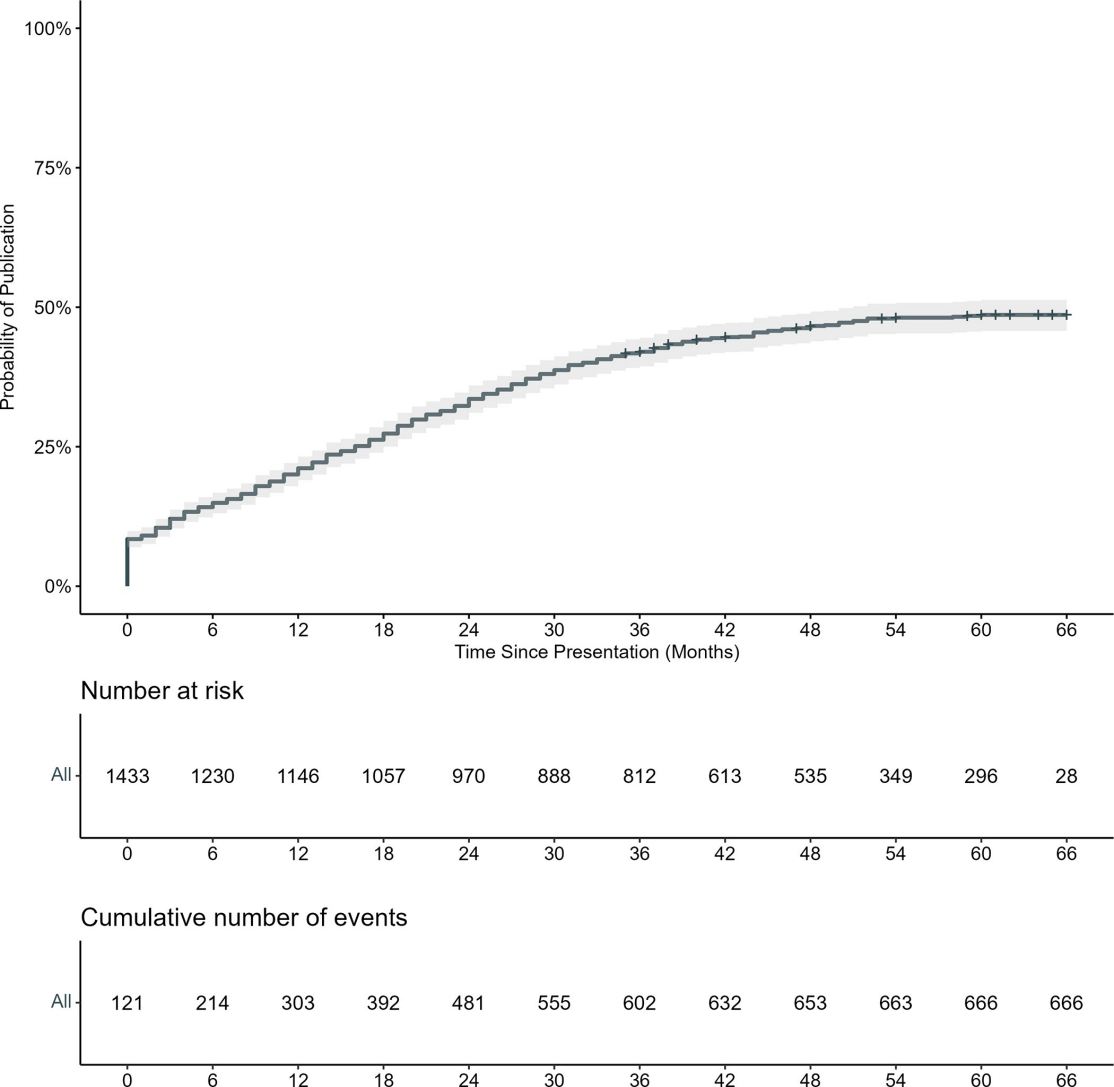
Kaplan-Meier estimate of time to publication from conference date for pediatric abstracts that reported research conducted in low- and middle-income countries.

In Kaplan-Meier analysis, the probability of publication of the results of studies presented as abstracts was 33.6% (95% confidence interval [CI] 31.2–36.1%) at 24 months and 46.6% (95% CI 44.0–49.3%) at 48 months (Fig 2). The probability of publication of pediatric global health abstracts was higher for abstracts that reported research conducted in several countries from multiple income brackets than those that reported research conducted in upper-middle income, lower-middle income, and low-income countries alone (S1 Fig).

In our multivariable Cox proportional hazard model, abstracts that reported research conducted in LMICs in East Asia and Pacific (adjusted hazard ratio [aHR] 3.06, 95% CI 1.79–5.24), South Asia (aHR 2.25, 95% CI 1.30–3.91%), and upper-middle-income countries (1.50, 95% CI 1.12–2.02) were published sooner than those that reported research in LMICs in Europe and Central Asia and lower-middle-income countries, respectively (Table 3 and S2 Fig). Conversely, abstracts that had 1–5 authors had a slower time to publication time than abstracts with >5 authors (aHR 0.80, 95% CI 0.67–0.96), and those that were abstracts only, that is without any indication of an accompanying poster or oral presentation (aHR 0.57, 95% CI 0.33–0.98%) were published later than those whose presentation type was unclear.

Compared to abstracts that contained results of cross-sectional studies, those that reported results from cohort studies (aHR 1.29, 95% CI 1.07–1.56) and randomized controlled trials (aHR 1.70, 95% CI 1.31–2.20) were published sooner. In contrast, abstracts that reported results from case series were published slower than cross-sectional studies (aHR 0.47, 95% CI 0.26–0.87).

## Discussion

In our study of 1,433 pediatric global health abstracts presented at seven major international conferences, fewer than half of the abstracts had accompanying peer-reviewed publications years after the conference. Abstracts that reported research on several infectious disease categories, including neglected tropical diseases and malaria, more commonly resulted in peer-reviewed publications. Independent determinants of faster time to publication for pediatric global health abstracts included study region, country income bracket, and study design.

Prior studies assessing nonpublication of pediatric abstracts have been limited to single conferences [16–18] and single study designs [19, 20]. Our study included abstracts from seven major conferences and all study designs, which provides a more comprehensive view of the scope of nonpublication rates. Publication rates for abstracts have ranged from as low as 11% in a pediatrics conference in Argentina to nearly 80% in pediatrics conferences in the United Kingdom and oncology conferences in the United States [17, 21, 22]. Our finding that 46.5% of pediatric global health abstracts had accompanying publications years later aligns with contemporary analyses of publication rates of abstracts in pediatric emergency medicine in the United States [10], orthopedic surgery in Australia [23], and post-graduate trainee abstracts in Pakistan [24]. To our knowledge, this is the first study to evaluate publication rates of pediatric global health abstracts. Nonpublication of abstracts is especially detrimental for the advancement of health of vulnerable populations such as children in resource-limited settings. As most investigators and policy makers seek evidence in peer-reviewed publications, nonpublication of presented abstracts can lead to missed propagation of knowledge, wasted resources in repeating studies which did not show any benefit or intervention, and worse yet, repeating studies which may have caused harm or unnecessary exposures repetition of studies that did not show benefit which may cause unnecessary harm to study participants and wasted time and resources in additional studies when abstracts showed no benefit of interventions [18, 19, 22].

Abstracts that reported research on several infectious diseases, including neglected tropical diseases and malaria, more commonly resulted in peer-reviewed publications in our study. This may be the result of global health originally being rooted in infectious diseases, which may have led to more established research programs and greater understanding of the importance of results dissemination in publications. Previous studies of publication rates of general infectious disease abstracts suggest that approximately one in three abstracts resulted in peer-reviewed publications [25]. Neglected tropical diseases and malaria affect billions of people worldwide [26], making clear the need for high quality research to reduce the disease burden of these infections.

Abstracts that reported research conducted in East Asia and the Pacific and South Asia were published sooner than studies conducted in other regions. Previous studies have similarly demonstrated variation in publication rates of abstracts by geographic region of the study country. Pingray et al. demonstrated that, in reproductive health research performed in LMICs, a majority of published studies reported research conducted in South Asia and East Asia and the Pacific [27]. The results of a Cochrane review by Scherer et al. suggest that publication rates may be higher among abstracts reporting research conducted in the United States and Europe compared to other geographic regions [9]. Though our study was not designed to elucidate reasons for nonpublication of pediatric global health abstracts, the variation in publication rates by region may be reflective of regional differences in academic priorities [28] or differential limitations in time dedicated to research due to competing clinical or administrative responsibilities [24].

In addition to study country region, we also found that country income bracket was associated with time to publication of pediatric global health abstracts. Specifically, abstracts that reported research conducted in multiple income brackets were published sooner in unadjusted analyses, and those conducted in upper-middle-income countries alone were 50% more likely to be published before those conducted in lower-middle-income countries in our multivariable Cox proportional hazard model. Shorter time to publication for studies conducted in multiple countries or upper-middle-income countries likely reflects differences in available resources to complete and publish research. Along with the substantial financial and time resources required to conduct research, many journals require article processing charges that can be several thousand US dollars in some journals. Such costs may hinder investigators who conduct studies in lower-income regions from being published given relative financial constraints [29]. Further research is warranted to better understand reasons for different rates of publication by study country income bracket.

Pediatric randomized controlled trials and cohort studies conducted in LMICs were published sooner than studies with less robust study designs such as case series studies conducted in LMICs. This finding is consistent with prior work that demonstrated abstracts that reported results from randomized-controlled trials were 50% more likely to be published than studies with other study designs [9]. Sooner publication of randomized-controlled trials as well as cohort studies may be due to more rigorous research standards and procedures and additional funding compared to other study designs. Additionally, we did not assess the directionality of findings in the included abstracts, that is the impact of negative findings on publication, which has been documented previously [30].

Lastly, we found that studies with more authors were more likely to be published than those with fewer authors. Number of authors is not commonly reported as a factor in time to abstract publication; however, studies have shown higher rates of publication for research with more authors as well as for multicenter versus single center studies [9]. Such multicenter research and research conducted in multiple countries may require the efforts of more investigators and, in turn, require the inclusion of more authors. Additionally, studies with more authors may be published sooner as there may be more individuals who contribute to research completion and manuscript writing.

### Limitations

Limitations of our study include the relatively short time from the conference to potential publication, with the minimum time prior to our review of around 2.5 years. Some studies presented as abstracts may have been ongoing during the conference presentation and could potentially be published at a later time after recruitment, data analysis, and manuscript preparation has completed. We could not account for the potential effect of the COVID-19 pandemic on the publication of results from abstracts presented prior to the pandemic. We attempted to include conference abstracts from conferences in diverse settings. However, the conference abstracts for conferences held in LMICs were not reliably available. Similarly, the conference proceedings were all published in English, making our results potentially not representative of conferences held in other languages. Although our publication search methods were standardized and used several databases, we may have missed some abstracts which were ultimately published or have been unable to reach or obtain a response from some authors to determine the status of any potential publications. Additionally, abstracts may have been improperly excluded or included based on the limited available abstract information, such as lack of clearly stating study population location.

## Conclusions

Fewer than half of pediatric global health abstracts were published in peer-reviewed journals up to four years after conference presentation. Several factors including more robust study designs and geographic study location were associated with sooner time to publication, suggesting a need to concentrate efforts to promote publication of research conducted in regions with low rates of publication. Efforts are urgently needed to promote the widespread and long-lasting dissemination of pediatric research conducted in LMICs presented as abstracts to provide a more robust evidence base for both clinical care and policy related to child health.

## Supporting information

S1 Fig Kaplan-Meier estimate of time to publication from conference date for pediatric global health abstracts by World Bank country income bracket. (TIFF)

S2 Fig Forest plot of factors associated with time to publication of pediatric global health abstracts. (TIFF)

S1 File STROBE checklist. (DOC)
