# Supplementary figures and images for: A cross-sectional analysis of publication of pediatric global health abstracts from seven major international conferences
Source: PLOS Glob Public Health. 2023 Oct 25;3(10):e0002523. doi: 10.1371/journal.pgph.0002523 (PMC10599509; doi:10.1371/journal.pgph.0002523)

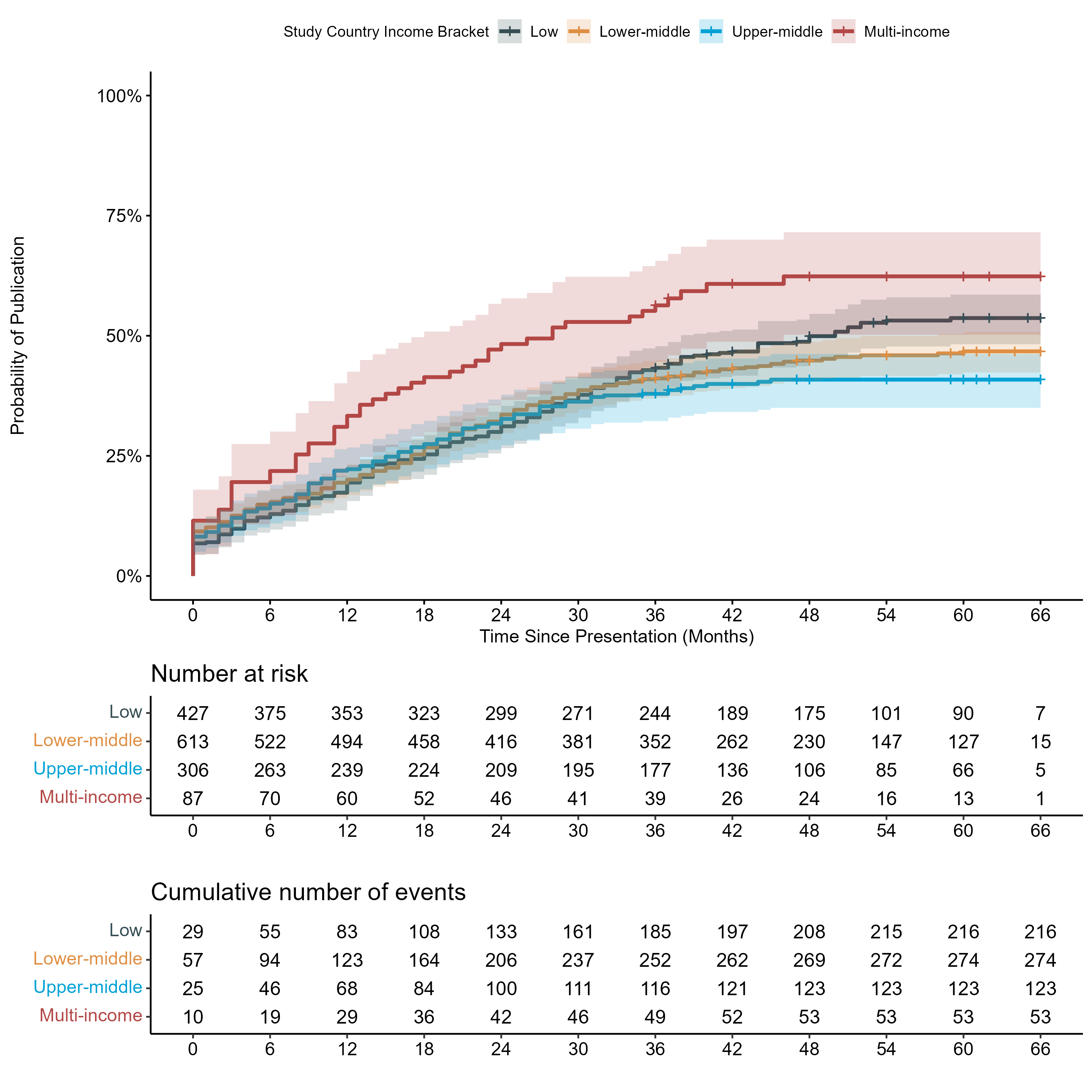

Supplement: S1 Fig — (TIFF) [file pgph.0002523.s001.tiff]
